# Pavlov's Cockroach: Classical Conditioning of Salivation in an Insect

**DOI:** 10.1371/journal.pone.0000529

**Published:** 2007-06-13

**Authors:** Hidehiro Watanabe, Makoto Mizunami

**Affiliations:** Graduate School of Life Sciences, Tohoku University, Sendai, Japan; University of Minnesota, United States of America

## Abstract

Secretion of saliva to aid swallowing and digestion is an important physiological function found in many vertebrates and invertebrates. Pavlov reported classical conditioning of salivation in dogs a century ago. Conditioning of salivation, however, has been so far reported only in dogs and humans, and its underlying neural mechanisms remain elusive because of the complexity of the mammalian brain. We previously reported that, in cockroaches *Periplaneta americana*, salivary neurons that control salivation exhibited increased responses to an odor after conditioning trials in which the odor was paired with sucrose solution. However, no direct evidence of conditioning of salivation was obtained. In this study, we investigated the effects of conditioning trials on the level of salivation. Untrained cockroaches exhibited salivary responses to sucrose solution applied to the mouth but not to peppermint or vanilla odor applied to an antenna. After differential conditioning trials in which an odor was paired with sucrose solution and another odor was presented without pairing with sucrose solution, sucrose-associated odor induced an increase in the level of salivation, but the odor presented alone did not. The conditioning effect lasted for one day after conditioning trials. This study demonstrates, for the first time, classical conditioning of salivation in species other than dogs and humans, thereby providing the first evidence of sophisticated neural control of autonomic function in insects. The results provide a useful model system for studying cellular basis of conditioning of salivation in the simpler nervous system of insects.

## Introduction

Secretion of saliva to aid in the initial mastication, digestion and swallowing of food is an important physiological function found in many vertebrates and invertebrates. Pavlov [1] reported classical conditioning of salivation in dogs about a century ago, and this form of conditioning is the best-known example of classical conditioning. Indeed, many of basic principles of classical conditioning have been established by studying this important form of learning. However, as far as we know, conditioning of salivation has been reported only in dogs and humans [2], [3]. In spite of the enormous effort by Pavlov and his successors [1], [4]–[6], its underlying neural mechanisms remain elusive because of the complexity of the mammalian brain.

Insects have been used as pertinent models in which to study the neural basis of learning and memory [7]–[10]. Studies using operant and classical conditioning procedures showed that cockroaches have excellent learning capabilities [11]–[15]. For example, cockroaches exhibited excellent learning performance in an occasion setting paradigm in which visual context defines the contingency between olfactory CSs (conditioning stimuli) and gustatory USs (unconditioned stimuli) [16].

Salivation is regulated by the autonomic nervous system. In mammals, salivary glands are supplied with chorinergic parasympathetic and adrenergic sympathetic nerves, which control secretion of protein-free and protein-rich saliva, respectively [17]–[19]. In insects such as cockroaches *Periplaneta americana*, salivary glands are supplied with dorpaminergic salivary neuron (SN1) and GABAnergic salivary neuron (SN2) of the suboesophageal ganglion and with several serotonergic neurons belonging to the stomatogastric nervous system [20]–[23]. Dopaminergic and serotonergic neurons control secretion of protein-free and protein-rich saliva, respectively [22].

We previously showed that salivary neurons of cockroaches exhibited a prominent response to an odor after conditioning trials in which the odor (CS) was paired with sucrose solution (US) but that these neurons exhibited only very weak responses to the odor presented alone without pairing with sucrose solution [24]. The results demonstrated conditioning of activity of salivary neurons, but direct evidence of conditioning of salivation remained to be obtained. In this study, we investigated the effects of conditioning trials on salivary responses to odors, and we found that an odor paired with sucrose solution induced an increase in the level of salivation. The results of this study demonstrate that classical conditioning of salivation is ubiquitous among different phyla.

## Methods

### Insects

Adult male cockroaches, *Periplaneta americana,* were obtained from a laboratory colony maintained under a light-dark cycle (LD 12:12) at 26–28°C. One or two weeks before the start of the experiment, a group of 20–30 cockroaches was placed in a chamber. In order to enhance motivation to uptake sucrose solution, cockroaches were fed a diet of sugar-free yeast extract and drinking water *ad libitum.*

### Measurement of salivation in response to olfactory or gustatory stimulus

The procedure for measurement of the level of salivation was modified from that used in our previous study [24]. A cockroach was briefly anaesthetized for restraining on a wax-coated dish ventral-side-up after its wings had been removed. The legs, neck and dorsal side of the abdomen were fixed with low-melting wax, and antennae were immobilized by staples. The restrained cockroach could move its mouthparts freely. Immobilized cockroaches were kept at room temperature for 1–2 hours.

**Figure 1:**
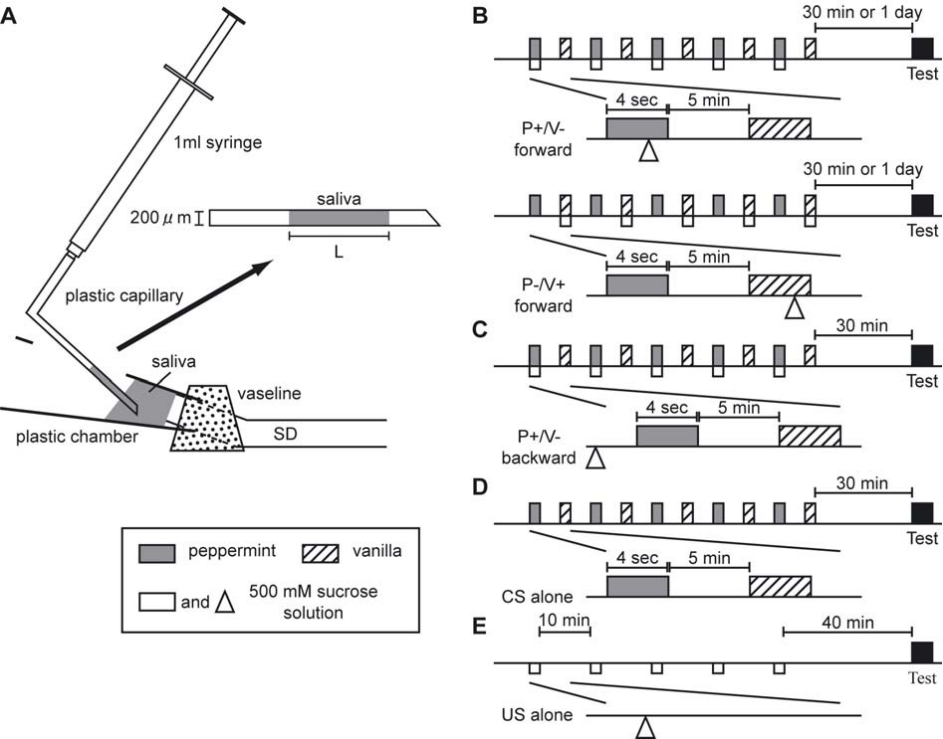
Experimental procedure. (A), Set-up for measurement of the amount of saliva secretion is illustrated as a lateral view. The distal cut-stump of the salivary duct (SD) was inserted into a small plastic chamber, and the tip of the plastic chamber was covered with white Vaseline (dotted square) to prevent leakage of saliva. Saliva (shaded area) secreted from the SD was collected by a plastic capillary attached to a syringe every 1 min. The amount of secreted fluid was calculated from the length (L) of the fluid column. (B), Stimulus schedules for five P+/V− or P−/V+ forward-pairing trials. One P+/V− or P−/V+ forward-pairing trial consisted of the presentation of peppermint (shaded rectangles) or vanilla odor (hatched rectangles) to an antenna before the presentation of sucrose solution (white squares and white triangles) to the mouth and presentation of vanilla or peppermint odor without pairing with sucrose solution, respectively. (C), Stimulus schedules for five P+/V− backward-pairing trials. One P+/V− backward-pairing trial consisted of the presentation of peppermint odor after the presentation of sucrose solution and subsequent unpaired presentation of vanilla odor. (D), Stimulus schedule for unpaired presentation of peppermint and vanilla odors (CS alone). Peppermint and vanilla odors were alternately presented five times without pairing with sucrose solution. (E), Stimulus schedules for unpaired presentation of sucrose solution (US alone). Sucrose solution was presented five times without pairing with an odor. The duration of olfactory stimulus was 4 sec. The inter-stimulus intervals were 5 min in B–D and 10 min in E. The measurements of salivation responses to peppermint, vanilla and apple odors (Test; black squares) were performed at 30 min (B–E) or 1 day (B) after conditioning or control trials.

Saliva is secreted into the oral cavity via the salivary duct. To expose the salivary duct, cuticles of the neck and labium were removed. The salivary duct was cut, and then the distal cut-stump was inserted into a plastic chamber with a hole in the upper part. The tip of the plastic chamber was covered with white Vaseline to prevent leakage of saliva (Fig. 1A). The measurement was initiated >10 min after completing the set-up of the preparation to stabilize salivation. Fluid secreted from the duct to the plastic chamber was drawn into a plastic capillary every 1 min, and the length of the fluid column was measured for calculating the volume of saliva (Fig. 1A). A level of salivation is continuously maintained in cockroaches [24].

### Sensory stimulation and conditioning procedure

The continuous airflow system used to deliver odor to an antenna was described previously [25]. In short, air passed through a small chamber containing a piece of filter paper was delivered to an antenna by operating a solenoid valve, without changing the flow rate. The filter paper was soaked with 40 µl of peppermint, vanilla or apple essence. The air around the antenna was continuously sucked out through a vacuum system. For gustatory stimulation, a droplet (4 µl) of 500 mM sucrose solution or 5 M sodium chloride solution was presented to the mouth.

The conditioning procedure was modified from that described previously [24]. Immobilized cockroaches received five P+/V− or P−/V+ forward-pairing trials (Fig. 1B) or P+/V− backward-pairing trials (Fig. 1C) with inter-trial intervals of 5 min. One P+/V− forward-pairing trial consisted of presentation of peppermint odor 3 sec prior to the presentation of sucrose solution and subsequent presentation of vanilla odor without pairing with sucrose solution (Fig. 1B). One P−/V+ forward-pairing trial consisted of unpaired presentation of peppermint odor and subsequent presentation of vanilla odor 3 sec prior to the presentation of sucrose solution. One P+/V− backward-pairing trial consisted of presentation of peppermint odor 3 sec after the presentation of sucrose solution and subsequent unpaired presentation of vanilla (Fig. 1C). In one control experiment (CS alone trials), peppermint and vanilla odor were alternately presented five times with intervals of 5 min (Fig. 1D). In another control experiment (US alone trials), sucrose solution was presented five times with intervals of 10 min (Fig. 1E). In all experiments, the duration of odor presentation was 4 sec.

To study the retention of the conditioning effect, salivation responses to odors were measured at 30 min after conditioning in some groups of cockroaches and at 1 day after conditioning in other groups. Cockroaches received 4-sec presentation of peppermint, vanilla and apple (control) odors with intervals of 6 min. The sequence of odor presentations was randomized among individuals, and data from the individuals were pooled for statistical evaluation. In experiments to study 1-day retention of the conditioning effect, immobilized cockroaches were kept in a moist chamber at room temperature.

## Results

### Salivation response to olfactory or gustatory stimulation

**Figure 2:**
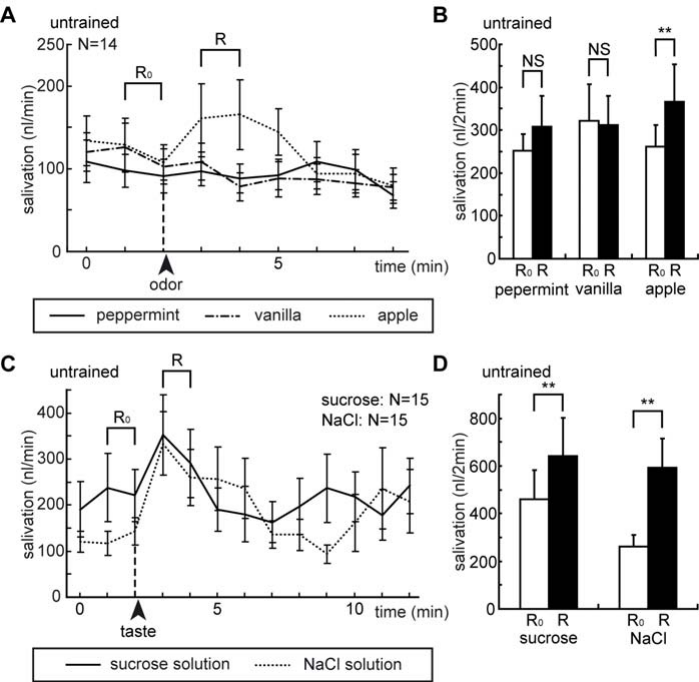
Changes in salivation levels upon olfactory (A,B) or gustatory (C,D) stimulations. In one group of cockroaches, peppermint (solid line), vanilla (broken line) and apple odors (dotted line) were presented for 4 sec to an antenna with intervals of 6 min (A). In another two groups of cockroaches, 4 µl of 500 mM sucrose solution (solid line) or 5 M sodium chloride solution (dotted line) was presented to the mouth (C). The amount of saliva secreted from the salivary duct was measured every 1 min, and values are shown as means±s.e.m. For statistical evaluation, the amount of saliva secreted for a 2-min period after the onset of olfactory (B) or gustatory (D) stimulation (R; black bar) was compared with that before stimulation (R_0_; white bar). Asterisks indicate the level of significance (** <0.01, NS>0.05; WCX-test).

Immobilized cockroaches exhibited steady levels of salivation of 100–200 nl/min, in accordance with our previous observation [24]. In one group of naïve (untrained) cockroaches (Fig. 2A,B), peppermint, vanilla and apple odors were applied to an antenna and resulting changes in the level of salivation were measured. In each of another two other groups of naïve cockroaches, salivation response to 500 mM sucrose solution or to 5 M sodium chloride solution applied to the mouth was measured (Fig. 2C,D), respectively. Apple odor (Fig. 2A), sucrose solution (Fig. 2C) and sodium chloride solution (Fig. 2C) induced an increase in the level of salivation for 2–3 min. In order to statistically evaluate salivation responses to olfactory or gustatory stimulations, the amount of saliva secreted during a 2-min period after the onset of stimulation (R) was compared with that during a 2-min period before stimulation (R_0_), using Wilcoxon's test. There were significant increases in the level of salivation in response to apple odor (Fig. 2B; N = 14, P<0.01, T = 8), sucrose solution (Fig. 2D; N = 15, P<0.05, T = 18) and sodium chloride solution (Fig. 2D; N = 15, P<0.01, T = 3) but not to peppermint (Fig. 2B; N = 14, P>0.05, T = 26) and vanilla odors (Fig. 2B; N = 14, P>0.05, T = 22). In the following conditioning experiments, we used peppermint or vanilla odor as conditioning stimulus (CS) and sucrose solution as unconditioned stimulus (US). Apple odor was used as a control odor. Sodium chloride solution was not used in subsequent experiments.

### Classical conditioning of salivation

**Figure 3:**
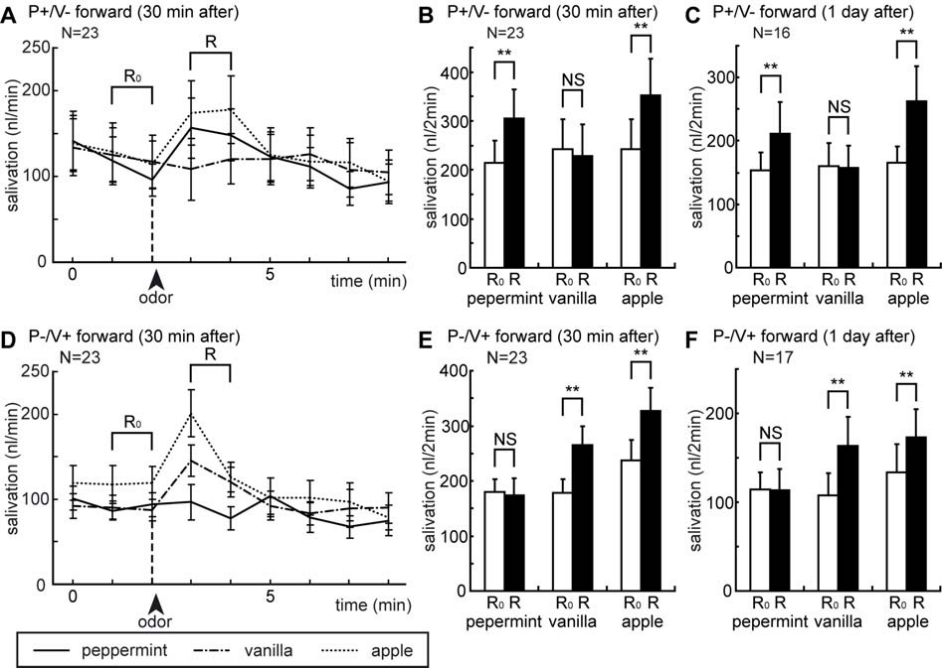
Changes in salivation levels in response to odors after P+/V− (A–C) or P−/V+ (D–F) forward-pairing trials. In one group of cockroaches, peppermint (solid line), vanilla (broken line) and apple odors (dotted line) were presented to an antenna at 30 min after five P+/V− (A) or P−/V+ (D) forward-pairing trials. The odors were presented for 4 sec with intervals of 6 min. The amount of saliva secreted from the salivary duct was measured every 1 min, and values are shown as means±s.e.m. For statistical evaluation, the amount of saliva secreted for a 2-min period after the onset of stimulation (R; black bar) was compared with that before stimulation (R_0_; white bar) at 30 min (B,E) or at 1 day (C,F) after five P+/V− or P−/V+ forward-pairing trials. Asterisks indicate the level of significance (** <0.01, NS>0.05; WCX-test).

Two groups of cockroaches were subjected to five sets of P+/V− conditioning trials, and changes in the levels of salivation in response to peppermint, vanilla or apple odor stimulation were measured at 30 min after conditioning trials in one group of cockroaches and at 1 day after conditioning trials in another group (Fig. 3A–C). Another two groups of cockroaches were subjected to five sets of P−/V+ forward-pairing trials, and salivation responses to these odors were measured at 30 min after conditioning trials in one group and at 1 day after conditioning trials in another group (Fig. 3D–F).

An increase in the level of salivation was observed when peppermint or vanilla odor was presented to an antenna at 30 min after P+/V− (Fig. 3A) or P−/V+ (Fig. 3D) forward-pairing trials, respectively. Increased levels of salivation lasted for about 2 min. To statistically evaluate salivation responses, the level of salivation for a 2-min period after odor stimulation was compared with that before odor stimulation. Peppermint or vanilla odor induced a significant increase in the levels of salivation at 30 min after P+/V− (Fig. 3B; N = 23; peppermint: P<0.01, T = 15) or P−/V+ (Fig. 3E; N = 23; vanilla: P<0.01, T = 27) forward-pairing trials, respectively. An increase in salivary responses to sucrose-associated odor was also observed at 1 day after P+/V− (Fig. 3C; N = 16; peppermint: P<0.01, T = 16) and P−/V+ (Fig. 3F; N = 17; vanilla: P<0.01, T = 6) forward-pairing trials, indicating that the memory lasted for at least 1 day.

Vanilla or peppermint odor presented alone without pairing with sucrose solution during P+/V− or P−/V+ forward-pairing trials induced no significant change in the level of salivation at 30 min and 1 day after P+/V− (Fig. 3B,C; vanilla; 30 min: N = 23; P>0.05, T = 115; 1 day: N = 16; P>0.05, T = 59.5) or P−/V+ (Fig. 3E,F; peppermint; 30 min: N = 23; P>0.05, T = 105; 1 day: N = 17; P>0.05, T = 73) forward-pairing trials, respectively.

### Control experiments

**Figure 4:**
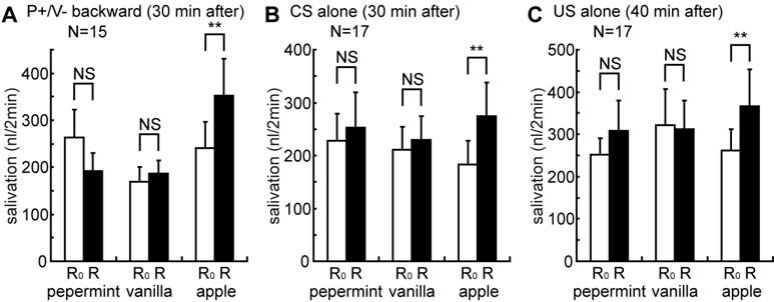
Changes in the levels of salivation in response to odors after control trials. In each of three groups of cockroaches, the amount of saliva secreted in response to peppermint, vanilla and apple odors was measured at 30 min after P+/V− backward (A), CS alone (B) or US alone (C) trials. The amounts of saliva were measured every 1 min, and values are shown as means±s.e.m. For statistical evaluation, the amount of saliva secreted for a 2-min period after the onset of olfactory stimulation (R; black bar) was compared with that before stimulation (R_0_; white bar). Asterisks indicate the level of significance (** <0.01, NS>0.05; WCX-test).

In three groups of cockroaches, changes in the levels of salivation in response to peppermint, vanilla or apple odor were measured at 30 min after P+/V− backward-pairing trials (Fig. 4A) or CS alone trials (Fig. 4B) or at 40 min after US alone trials (Fig. 4C). Salivation responses to odor stimulations were statistically evaluated by comparing the amounts of saliva secreted during a 2-min period after odor stimulation (R) with that before stimulation (R_0_), by Wilcoxon's test.

Peppermint or vanilla odor induced no change in salivation level in any of the three control groups, namely, P+/V− backward-pairing group (N = 15; peppermint: P>0.05, T = 29; vanilla: P>0.05, T = 48), CS alone group (N = 17; peppermint: P>0.05, T = 70; vanilla: P>0.05, T = 65) and US alone group (N = 17; peppermint: P>0.05, T = 60.5; vanilla: P>0.05, T = 69). Apple odor induced a significant increase in the level of salivation in all control groups (P+/V− backward-pairing group, N = 15; P<0.01, T = 10; CS alone group, N = 17; P<0.01, T = 15; US alone group, N = 17; P<0.01, T = 16), thereby indicating that the preparations remained intact.

## Discussion

Classical conditioning of salivation was first discovered in dogs by Pavlov [1]. By studying this important form of learning, he established the basis for modern scientific research on learning and memory. Subsequent studies successfully demonstrated conditioning of salivation in humans [2]–[3], [26], but as far as we know, this form of conditioning has not been reported in any other species.

In this study, we showed that (1) untrained cockroaches exhibited no salivation responses to vanilla or peppermint odor, (2) after differential conditioning trials in which an odor was paired with sucrose solution and another odor was presented alone, cockroaches exhibited salivation response to sucrose-associated odor but not to the odor presented alone, (3) the conditioning effect was maintained for 1 day after conditioning and (4) backward-pairing, CS alone and US alone trials did not induce a conditioning effect. The results demonstrate conditioning of salivation in cockroaches, for the first time in species other than dogs and humans, thereby demonstrating that conditioning of salivation is ubiquitous among different phyla.

Pavlov [1] reported that a lesion of the cerebral cortex decreased salivation in response to auditory conditioned stimulus in dogs. Subsequent studies in dogs suggested that the orbital cortex plays a role in modulation of salivation in response to auditory conditioned stimulus [4]–[6]. However, neural mechanisms underlying conditioning of salivation remain elusive because of the complexity of information processing in the mammalian brain. Cockroaches provide a useful set up to study neural mechanisms underlying conditioning of salivation since their brains consist of a relatively small number of neurons, many of which are individually identifiable in terms of their morphology and physiology [25], [27]–[29].

This study is also the first to demonstrate conditioning of autonomic function in invertebrates. Conditioning of autonomic function is demonstrated in mammals, birds and fishes [30], [31], but in invertebrates, previous reports on experience-dependent changes in autonomic function were limited to sensitization of heart rate in the mollusc *Aplysia* [32] and habituation of cardiac response to visual stimuli in the fly *Calliphora* [33] and the crab *Chasmagnathus* [34]. Our finding demonstrates that sophisticated neural control of autonomic function is not specific to vertebrates but applicable to insects.

In cockroaches, salivation is regulated by activities of salivary neurons and neurons of the stomatogastric nervous system [20], [23], and we previously reported that salivary neurons responded to gustatory stimuli. Salivary neurons of untrained cockroaches exhibited (1) a level of spontaneous activity, (2) strong responses to sucrose or sodium chloride solution applied to the mouth and (3) very weak responses to peppermint or vanilla odors applied to an antenna [24]. These features of activities of salivary neurons are in accordance with the features found for salivation, in that (1) there was a spontaneous level of salivation and (2) sucrose or sodium chloride solution induced an increase in the level of salivation but (3) peppermint or vanilla odor did not. For the last point, it is obvious that the responses of salivary neurons of untrained cockroaches to peppermint and vanilla odors are not strong enough to induce a significant increase in the level of salivation. Thus, we conclude that there is a good correlation between the activities of salivary neurons and the levels of salivation.

We also previously examined the effect of conditioning on odor responses of salivary neurons, and found that (1) an odor induced prominent responses in salivary neurons after being paired with sucrose solution, (2) the conditioning effect lasted for one day and (3) backward-pairing, CS alone and US alone trials were not effective in achieving conditioning of activities of salivary neurons [24]. These observations are in accordance with findings on the level of salivation in this study. Moreover, our study on the change in the level of salivation in response to electrical stimulation of salivary neurons suggested that the magnitude of increase in odor responses of salivary neurons is sufficient to lead to an increased level of salivation [24], indicating a causal relationship between the activities of salivary neurons and salivation. Therefore, we conclude that activities of salivary neurons offer a useful monitor of conditioning of salivation. We have not yet clarified whether the activities of neurons of the stomatogastric nervous system also exhibit a conditioning effect.

Conditioning of salivation should provide useful model system for studying neural basis of learning and memory in insects. In insects, most successful electrophysiological studies in search for neural correlates of learning and memory have been performed by using classical conditioning of proboscis extension response, in which an odor was associated with sucrose solution and activities of an extensor muscle of the proboscis were recorded as a measure of the conditioning effect [honeybees: 35,36; moths: 37]. One of difficulties of this experimental paradigm, however, is that the contraction of the proboscis extensor muscle often induces a movement of the brain, which prevents stable electrophysiological recordings of the activities of brain neurons. Classical conditioning of salivation in cockroaches provides an excellent alternative experimental system since conditioning of salivation can be easily monitored by extracellular recordings of activities of salivary neurons and, more importantly, activities of these neurons induce no movement of the brain. Moreover, stable intracellular recordings from brain neurons for >15 min are feasible in cockroaches [38]–[40]. The objective of our next experiment is to study the areas of the brain and the suboesophageal ganglion participating in conditioning of salivation.
